# Association between omega-3 index and depersonalization among healthcare workers in a university hospital: a cross-sectional study

**DOI:** 10.3389/fpsyt.2024.1425792

**Published:** 2024-11-15

**Authors:** Helena Sofia Antao, Ema Sacadura-Leite, Pedro Aguiar, Carlos Gois, Jessica Marques, Samuel Pombo, Maria Luisa Figueira

**Affiliations:** ^1^ Faculty of Medicine, University of Lisbon, Lisbon, Portugal; ^2^ Public Health Research Center (PHRC), National School of Public Heath, Lisbon, Portugal; ^3^ Occupational Health Department, Santa Maria Local Health Unit, Lisbon, Portugal; ^4^ Comprehensive Health Resource Center (CHRC), NOVA University of, Lisbon, Portugal; ^5^ Biostatistics, Amadora/Sintra Local Health Unit, Amadora, Portugal; ^6^ Psychology and Psychiatry Clinic, Faculty of Medicine, University of Lisbon, Lisbon, Portugal; ^7^ Department of Psychiatry and Mental Health, Santa Maria Local Health Unit, Lisbon, Portugal

**Keywords:** omega-3 index, burnout, biological correlate, depersonalization, risk factor

## Abstract

**Introduction:**

Burnout harms workers physical and mental health due to induced brain changes, autonomous nervous system and hypothalamus-pituitary-adrenal axis excessive activation. Although several correlations and risk factors have been identified, the research around burnout biological correlates remains underdeveloped. The omega-3 index has been proposed in mental health as a contributor to identify high risk patients and monitor disease advancements but the evidence on its relationship with burnout is limited. This study is meant to test the hypothesis that the omega-3 index is inversely associated with burnout levels and to discuss its potential as a biological correlate of burnout.

**Methods:**

It had an observational, cross-sectional design and was carried out at a university hospital center between March 2021 and July 2023. We invited 319 healthcare professionals (doctors and nurses) at the occupational health and emergency departments. The omega- 3 index was determined through a prick finger test. Emotional exhaustion, depersonalization and personal accomplishment were measured by the Maslach Burnout Inventory. Descriptive analyses were conducted to examine the participants’ characteristics and outcome variables. Means, medians, interquartile ranges and standard deviations were calculated for continuous variables. Frequencies and percentages were obtained for categorical variables. We have used the individual dimensions’ scores as continuous data in the evaluation of their relationship with the omega-3 index. The relationship between burnout levels and the omega-3 index was assessed through linear regression analysis.

**Results:**

We surveyed 300 subjects (94% response rate). High emotional exhaustion and depersonalization were reported by 29.9% and 22.1% of participants, respectively; 26.0% reported low personal accomplishment. The mean omega-3 index was 5.75%. The depersonalization score was, on average, 11.132 points higher (95% CI [4.661; 17.603]) in individuals whose omega-3 index was lower than 4%.

**Discussion:**

An omega-3 index below 4% seems to potentially be a biological correlate of depersonalization. Our results contribute to enlarging the knowledge about burnout biological correlates, an area that has been previously signalled as underdeveloped. Omega-3 index should be included in prospective studies that will investigate the evolution of other burnout biological correlates as the syndrome emerges and progresses in subjects at risk.

## 1 Introduction

Burnout has been conceptualized by Maslach et al. (1) as a prolonged response to chronic emotional and job-related stressors and is recognized to be one of the most relevant psychosocial occupational risks of contemporary societies. It initially described symptoms of emotional fatigue, loss of dedication and motivation experienced mainly in care providers and person-oriented jobs (healthcare professionals, teachers, police officers, social workers), where bureaucratization and lack of reciprocity often replace the workers’ initial idealism by feelings of frustration and disillusion (1, 2), but it is now known that burnout can affect people from all occupations. The three core dimensions of burnout are emotional exhaustion (EE), depersonalization (DP) and reduced personal accomplishment (PA). EE is the experience of being drained and depleted by one’s workload; DP is described as a defensive coping strategy where individuals limit involvement with others and create distance and indifference from their work; lack of PA reflects the feeling of not performing tasks adequately and a tendency of evaluating own’s results negatively (3–5). Excessive job demands, monotonous functions, conflict with managers and peers as well as confrontational customers’ behavior have been described as risk factors for burnout (3).

Although several other measurement instruments have been developed and applied in research [e.g. the Burnout Assessment Tool (6), the Oldenburg Burnout Inventory (7) and the Copenhagen Burnout Inventory (8)], the Maslach Burnout Inventory (MBI) remains the most widely used.

It is universally accepted that burnout may result not only in harmful consequences for workers health but also in decreased quality of customer care. Uncontrollable stress reduces prefrontal cortex activity which affects cognition and regulation of emotions. This in turn may lead to reduced motivation, unprofessional behavior and ineffective communication with customers (9). This explains why researchers, organizations and public health specialists dedicate so much attention to understanding the problem and finding effective solutions to workers’ burnout.

Although burnout is widely accepted as a work-related syndrome emanating from workers’ exposure to long-term stressors with which they miss to cope, there is consensus that affected people need medical treatment and psychological support. For this reason, there is a never-ending debate about whether burnout should be recognized as a clear psychiatric disorder with its own diagnosis, or a side construct of others e.g., major depression, adjustment disorder, generalized anxiety or chronic fatigue syndrome (10–12). Notably, its overlap with depression has been subject to detailed analysis and discussion (13–16), supporting the hypothesis of a partially shared neurobiological basis. Although the Diagnostic and Statistical Manual of Mental Disorders in its fifth edition (DSM-5) (17) does not describe burnout as a mental disorder, the International Classification of Diseases in its eleventh edition (ICD-11) defines it as a syndrome resulting from chronic workplace stress and states it should not be considered if adjustment disorders, disorders specifically associated with stress, anxiety, fear-related or mood disorders are present (18). Healthcare workers are a high-risk group for burnout worldwide (19, 20). The prevalence rates of burnout among healthcare providers reported in a profusion of studies carried out in diverse locations show significant variation (21–23, 43). Rotenstein et al. (24) conducted a systematic review of published articles on physician burnout, revealing overall prevalence rates ranging from 0% to 80.5%. These differences may be due to the variability in source populations (e.g. age, job tenure, residency status, weight of frontline and emergency participants), measurement instruments, efficacy of in place occupational health programs, variability in training and psychological support provided to workers (16).

Healthcare workers’ burnout levels and other aspects of mental health were profoundly affected by COVID-19 (25–30). Pandemic situations are long known to increase the risk of adverse mental effects in these workers (42), which may be due to higher exposure to contagion (31), increased workload (32), frequent onsite redeployment as well as constantly changing guidelines in patients’ management and other working procedures (33).

Research carried out during the COVID-19 pandemic identified specific factors that could additionally increase the risk of burnout amongst doctors and nurses as decreased social support, low family and colleagues’ readiness to cope with the outbreak, frontline work, being a resident doctor, death of patients under one’s care and low level of specialized training (34–36, 43).

On the other hand, some protective factors have also been identified both before and during the outbreak such as hardiness, resilience, self-efficacy, job satisfaction, satisfaction with life, adequate sleeping hours and days off, perceived safety conditions, working environment, coping strategies, patients’ support and gratitude, payment and policies governing professional practice (37–43).

Burnout in nurses and physicians affects the quality of patient care, resulting in lower patient satisfaction and a higher number of medical errors (44–47). Hodkinson et al. (48) reported that physicians burnout can double the risk of patient safety and contribute to 7% to 10.6% of serious medical mistakes.

Burnout is also harmful to those it affects. It damages workers mental health, which is reflected in higher incidences of anxiety, depression, post-traumatic stress disorder and insomnia (49). A higher probability of physical health problems has also been associated with burnout, e.g. hypertension, type II diabetes mellitus, cardiovascular disease, musculoskeletal pain, gastric disease, chronic headaches, decreased immune response, chronic fatigue, and insomnia (50).

Specifically, in healthcare providers, burnout has been linked to increased rates of depression, anxiety and suicidality (51), to positive associations with metabolic syndrome (52), as well as to a higher cardiovascular risk (53) and hyperlipidemia (54).

The underlying biological hypothesis for burnout harmful effects on overall health is that all directly or indirectly result from excessive and prolonged stress-related activation of the autonomous nervous system and of the hypothalamus-pituitary-adrenal axis, that prevent these systems to return to homeostasis (55–57).

Brain changes have also been described in animal models and human imaging studies including impaired neurogenesis in hippocampal and frontal structures, thinning of medial frontal cortex, bilateral increase of amygdala volumes as well as grey matter reductions in the anterior cingulate cortex, the dorsolateral prefrontal cortex, the caudate and the putamen nucleus (58).

Several potential burnout biomarkers have been studied to date, among which markers of stress (cortisol, α-amylase, serotonin), cytokines (e.g. IL-6, TNF-α, IL-β), platelet-to-lymphocyte ratio, C reactive protein, prolactin, melatonin, adiponectin, total cholesterol, and cholesterol fractions (59, 61, 69). Some authors have used the term “allostatic load” to describe the sub-clinical accumulated physiological dysregulation that results from exposure to chronic stressors and identified a number of physiological indicators including systolic and diastolic blood pressure, high-density lipoprotein and total cholesterol, fasting plasma glucose, glycosylated hemoglobin, serum dehydroepiandrosterone, plasma cortisol or 24-hour urinary cortisol, adrenalin excretions, body mass index and waist circumference (60–62).

Although multiple correlations and risk factors were found, differences in methodologies and definitions make comparisons across studies difficult to make and have so far hampered definite conclusions. This research area remains underdeveloped as previously pinpointed by other authors (4).

The n-3 fatty acids (n-3 FA) supply to the human body is achieved through diet, especially fatty fish and flaxseed. Their blood levels, metabolism and absorption depend on individual conditions, age and genetic factors (63–65).

The knowledge on n-3 FA impact on the central nervous system (CNS) arises mainly from studies carried out in animal studies and clinical trials that studied their effect on psychiatric illnesses. They influence CNS health through anti-inflammatory and neuromodulation mechanisms. Their presence in membranes modulates cytokine release and perfusion (66), downregulating inflammation in psychiatric diseases as depression, stress disorders, schizophrenia, and dementia (67–69). As part of CNS’ membranes phospholipid acyl chains, n-3 FA are also pivotal to their fluidity, ion exchange, signaling and neurotransmission (70, 71). The improvement of mood disorders achieved through high ingestion of n-3 FA may stem from the modulation of proteins that take part in brain perfusion and neurotransmitter’s uptake (especially serotonin and dopamine) (72–74). In dementia, where the accumulation of amyloid beta (Aβ) results in synaptic loss, tau protein hyperphosphorylation and formation of neurofibrillary tangles (75), n-3 FA have shown to dampen oxidative stress and inflammation (76).

The omega-3 index (O3I) is defined as the red-blood cell membrane’ percentage of eicosapentaenoic acid (EPA) and docosahexaenoic acid (DHA). A large body of research has shown that O3I reflects tissues’ content in EPA and DHA (77). It is accepted as a reliable long-term marker of n-3 FA intake and has shown to independently deliver predictive information for several illnesses (78), including but not limited to mental disorders. O3I levels have been categorized as desirable (>8%), moderate (>6 to 8%), low (>4 to 6%), or very low (≤4%) by researchers who analyzed their overall impact in human health (79, 80).

Although no target O3I has so far gathered consensus in the field of mental health, several studies have described relationships between O3I and mental illnesses. Some authors have proposed the concept of an O3I in mental health that could help physicians in identifying high risk patients and in monitoring disease advancements (81–83). A recent review (16) suggested that O3I may be considered a risk factor for some psychiatric diseases and proposed risk thresholds of 4–5% in major depression and dementia, 5% in postpartum depression and 4% for psychosis transition. One study has assessed O3I before and after effective psychological treatment in soldiers suffering from Post Traumatic Stress Disorder (84) and showed a significant O3I increase in treated patients which concedes that O3I varies with disease severity and may also be a marker of non-dietary treatment effectiveness.

To our knowledge, O3I has not been studied before as a potential biological correlate of occupational burnout.

Burnout scores have been assessed before and after supplementation with n-3 FA in two placebo-controlled clinical trials. One of them found improvements in all three dimensions of burnout, measured through the MBI, after eight weeks of supplementation (23) but did not evaluate the participants’ O3I and therefore no conclusions can be drawn on that relationship. The other described a significant increase in O3I after twelve weeks, but no difference was found in burnout scores measured through the Copenhagen Burnout Inventory between the active and the control groups (85).

The paucity and blurriness of evidence concerning O3I relationships with burnout is not surprising having in mind the biomarkers research underdevelopment that other authors have highlighted (4).

The n-3 FA influence on inflammation, oxidative stress and brain structure described earlier, along with the hypothetical biological mechanisms of burnout favors our conjecture that O3I may be associated with burnout severity.

This study is meant to test the hypothesis that O3I is inversely associated with burnout levels and to discuss its potential as a biological correlate of burnout.

## 2 Materials and methods

### 2.1 Study design and source population

This was an observational, cross-sectional study, with data collected in two periods between March 2021 and July 2023 (first period: March 2021 – June 2021, second period: October 2022 – July 2023) at the occupational health and emergency departments of a university hospital center located in Lisbon, Portugal.

The sample size was determined with the support of the Raosoft^®^ sample size calculator (86) and fixed at 300 participants considering a maximum burnout prevalence of 33% (which was the high emotional exhaustion rate found in the first 106 participants), an acceptable error margin near 5% and a 95% confidence level.

The study used a convenience sampling method at both recruitment sites. At the Occupational Health department, researchers invited in person doctors and nurses who attended admission, periodic and occasional work medicine appointments. At the Emergency department, doctors and nurses were approached in person at their workstations. To be eligible to participate, subjects had to be doctors or nurses with an active work contract signed with the hospital. All participants were invited in person. None were asked to provide explanations if refused to participate. Those who refused to participate spontaneously alleging lack of time were informed of the next available dates for participation; at the Emergency department the researcher also went back half an hour to one hour later for a second attempt. As an incentive to participate, subjects were offered the possibility of getting their omega-3 index report by electronic mail. The 300 doctors and nurses who agreed to participate in the study were surveyed after providing written informed consent.

### 2.2 Variables and instruments of measurement

Questionnaires and blood collection were performed by trained researchers.

The questionnaire included items about demographics and other individual characteristics (including age, gender, professional category, job tenure, department of origin, specialty when applicable and intake of food supplements).

Diet EPA and diet DHA are the monthly seafood intake of EPA and DHA, expressed in grams, collected by means of a food frequency questionnaire. Calculations were based on EPA and DHA contents of each species (87–91).

#### 2.2.1 Maslach Burnout Inventory – Human Services Survey

To determine burnout levels, participants filled in the Portuguese validated version (92) of the 22 items Maslach Burnout Inventory – Human Services Survey (MBI-HSS).

MBI-HSS is the most widely used instrument amongst healthcare workers. It has been designed for professionals working in human services (nurses, physicians, social workers, health counsellors, therapists, clergy, police and correctional officers) and other jobs focused on helping people by providing guidance, protection, and improving physical, emotional, or cognitive problems (1). The MBI-HSS addresses three scales: EE, which measures feelings of being emotionally overextended and exhausted by one’s work; DP, which measures an unfeeling and impersonal response toward recipients of services, care or instruction; PA, which measures feelings of competence and successful achievement in one’s work.

In the definition of low, average and high levels of each dimension of the MBI-HSS, we have used the following cut-offs: EE: low, ≤13; average, 14-26; high, ≥27; DP: low, ≤5; average, 6-9; high, ≥10; PA: low, ≤33; average, 34-39; high, ≥40 (93).

#### 2.2.2 Mini International Neuropsychiatric Interview (MINI)

All participants were assessed through the Mini International Neuropsychiatric Interview (MINI) (94, 95), a brief structured diagnostic interview that assesses the most common disorders in mental health. This questionnaire was included to allow controlling the results for the variable “current depressive episode”.

#### 2.2.3 Omega-3 prick finger test

Participants O3I was determined through an omega-3 prick finger test (Omegaquant®). In this test, red blood cells composition is analyzed according to the HS-Omega-3 Index methodology (96, 97). Tests’ codes were registered in a database provided by the supplier and accessed by the main researcher at the end of the study.

### 2.3 Statistical analysis

Descriptive analyses were conducted to examine the participants’ characteristics and outcome variables. Means, medians, interquartile ranges and standard deviations were calculated for continuous variables. Frequencies and percentages were obtained for categorical variables.

We have used the individual dimensions’ scores as continuous data in the evaluation of their relationship with O3I (12).

To determine potential risk or protection O3I thresholds we have tested several cut-offs: O3I <3%, O3I <4%, O3I <5%, O3I ≥7%, O3I ≥8% and O3I ≥9%.

This relationship was assessed through linear regression analysis and adjusted to variables as recommended by other authors due to their potential influence in either burnout or O3I: age (75), current depressive episode (25), omega-3 supplements intake and EPA/DHA diet content (98). As job tenure and age are co-variates (99) we have chosen an adjustment to age because there is evidence of interactions between this variable and omega-3 body levels. We have applied the Bonferroni correction to adjust significant p-values when testing multiple O3I thresholds.

To confirm normality, linearity, homoscedasticity, and absence of multicollinearity we have carried out residual plots and variance inflation factor analysis.

We have used Spearman coefficients to determine correlations amongst EE, DP and PA in order to ensure internal data validation.

All reported p values are two-tailed and p < 0.05 was considered statistically significant.

All analyses were performed using IBM SPSS Statistics v.26.

### 2.4 Ethics statement

This study was approved by Comissão de Ética do CHULN e CAML (Ethics Committee of Centro Hospitalar e Universitário de Lisboa Norte and of Centro Académico de Medicina de Lisboa), registration number 305/20, July 20th, 2020.

## 3 Results

Of the 319 healthcare workers invited, 300 accepted to participate (94% response percentage) of which 167 doctors and 133 nurses. Two blood samples did not meet the quality criteria for analysis. Valid samples from 298 participants were considered for analysis involving n3-FA values, ratios and associations with burnout.

The full list of medical specialties and departments is provided in a **Supplementary Data Sheet 1**.

High EE and DP scores were reported by 29.9% and 22.1%, respectively; 26.0% reported a low PA score.

DP showed a positive correlation with EE and a negative correlation with PA. EE was also negatively correlated with PA. Although weak to moderate, all correlations found amongst the three dimensions were statistically significant.

The mean O3I of this sample was 5.75%; 4.7% and 2.7% of participants were in the very low (O3I <4%) and desirable (O3I≥8%) O3I ranges, respectively.

**Table 1** summarizes the main participants’ sociodemographic characteristics, O3I values and burnout rates.

**Table 1:** Sociodemographic characteristics and burnout rates (n=300).

**Age, median, IQR**	36.57 (21.0, 68.0)
Gender, n (%)	
Male	85 (28.3%)
Female	215 (71.7%)
Professional category, n° (%)	
Nurses	133 (44.3%)
Doctors	167 (55.7%)
**Job tenure in years, median, IQR**	10.23 (0.0, 43.0)
**Current depressive episode, n (%)**	21 (7.0%)
Omega-3 index	
Mean, %	5.75
Median (IQR), %	5.59 (3.03, 9.01)
< 4%, n (%)	14 (4.7%)
≥ 4% and <8%, n (%)	276 (92.5)
≥ 8%, n (%)	8 (2.7%)
Diet EPA, g/month	
Median (IQR)	5.586 (0.0, 29.89)
Diet DHA, g/month	
Median, IQR	11.964 (0.0, 62.13)
**Omega-3 supplementation, n (%)**	14 (4.7%)
Burnout Status	
Emotional Exhaustion (n=291)	
Mean	21.05
Median (IQR)	21.00 (0.0, 53.0)
% High	87 (29.9%)
% Average	123 (42.3%)
% Low	81 (27.8%)
Depersonalization (n=298)	
Mean	6.03
Median (IQR)	5.00 (0.0, 27.0)
% High	66 (22.1%)
% Average	69 (23.2%)
% Low	163 (54.7%)
Personal Accomplishment (n=296)	
Mean	37.08
Median (IQR)	38.00 (0.0, 48.0)
% High	116 (39.2%)
% Average	103 (34.8%)
% Low	77 (26.0%)

**Figure 1:**
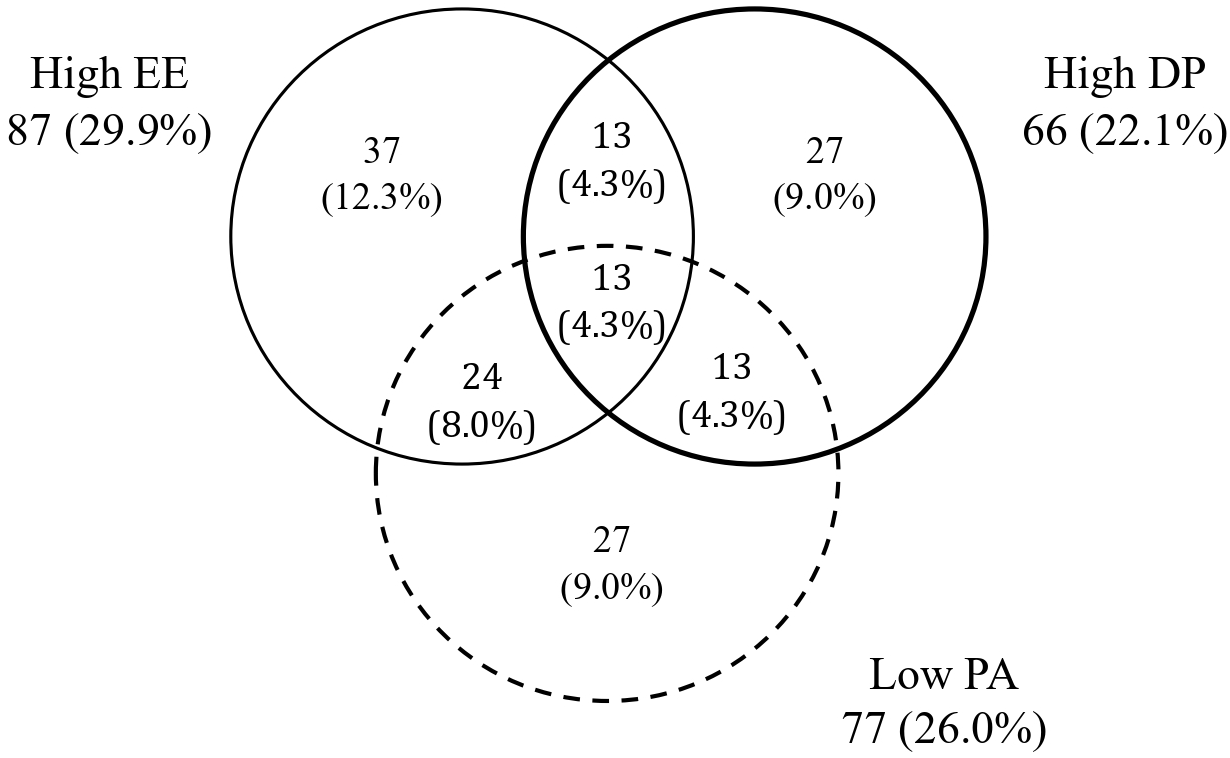
Venn's diagram representing the number of participants with high burnout scores in each of the three dimensions (n = 300) Results are expressed as number (percentage) of subjects. EE, emotional exhaustion; DP, depersonalization; PA, personal accomplishment.

**Figure 1** depicts the number and percentages of participants with high burnout scores in each of the three dimensions and related overlaps.

**Figure 2:**
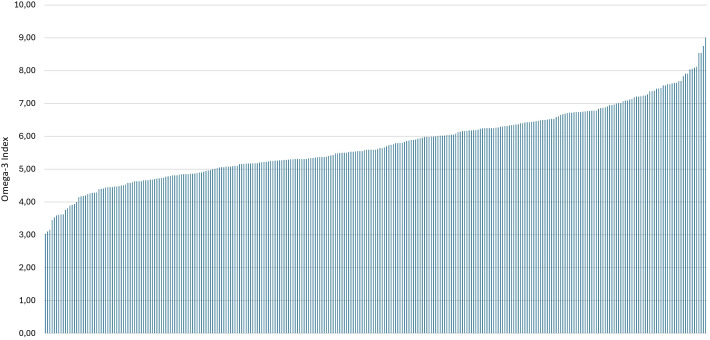
Individual O3I determined in valid study samples (n = 298).

**Figure 2** portrays the individual O3I distribution across the sample.

O3I <4% was associated with DP; DP score was, in average, 11.132 points higher (95% CI [4.661; 17.603]) in individuals whose O3I was below 4% than in individuals whose O3I was 4% or higher.

No O3I relationships with EE or PA were found. **Table 2** illustrates the associations between the three burnout dimensions analyzed as continuous variables and O3I as continuous and categorical variable, considering several O3I cut-offs. **Table 3** outlines the Spearman’s correlation coefficients amongst the burnout dimensions.

**Table 2:** O3I variables related to burnout dimensions on linear regression analysis controlled by age, current depressive episode, DHA/EPA diet content and omega-3 supplementation.

	Emotional Exhaustion (n=290)			Depersonalization (n=297)			Personal Accomplishment (n=295)		
	adjusted β	C.I.	p value	adjusted β	C.I.	p value	adjusted β	C.I.	p value
**O3I** (quantitative)	0.328	[-1.354; 2.010]	0.700	-0.333	[-1.073; 0.406]	0.374	-0.139	[-1.151; 0.874]	0.787
**O3I < 3%**	–	–	–	–	–	–	–	–	–
**O3I < 4%**	-8.215	[-23.141; 6.711]	0.278	**11.132**	**[4.661; 17.603]**	<0.005*	4.068	[-4.945; 13.082]	0.374
**O3I < 5%**	-1.331	[-5.949; 3.288]	0.570	0.087	[-1.958; 2.132]	0.933	0.008	[-2.866; 2.883]	0.995
**O3I ≥ 7%**	-0.751	[-5.448; 3.946]	0.752	-1.752	[-3.819; 0.314]	0.096	1.059	[-1.738; 3.856]	0.455
**O3I ≥ 8%**	-1.744	[-10.185; 6.696]	0.683	-0.906	[-4.714; 2.902]	0.639	-0.480	[-5.590; 4.631]	0.853
**O3I ≥ 9%**	6.397	[-13.85; 26.64]	0.533	6.196	[-2.901; 15.292]	0.180	-4.094	[-16.353; 8.166]	0.510

*Bonferroni correction applied.

Statistically significant values in bold.

**Table 3:** Spearman’s correlation coefficient (rs) amongst burnout dimensions.

		Emotional Exhaustion	Depersonalization	Personal Accomplishment
**Emotional Exhaustion**	rs	1.000	0.308	-0.376
	p value	–	<0.001	<0.001
**Depersonalization**	rs	0.308	1.000	-0.347
	p value	<0.001	–	<0.001
**Personal Accomplishment**	rs	-0.376	-0.347	1.000
	p value	<0.001	<0.001	–

Residual plots and variance inflation factor analysis are provided as **Supplementary Material**.

## 4 Discussion

Our study participants had on average an O3I of 5.75% which is less than desirable and is categorized as a low n-3 bio-status. Although this value is within the expected range for a European country (91, 92), higher levels would be anticipated in participants living in a country that ranks six in the list of world fish and seafood consumption per capita (100). It is also lower than the average O3I reported in a study carried out in the same country amongst elderly users of a primary care setting (101). This finding is consistent with the evidence that a variety of health states may influence the n-3 FA individual status and that O3I is more than a consequence of dietary intake.

The prevalence of high burnout levels found in our study (29.9% for high EE, 22.1% for high DP and 26.0%. for low PA). These figures are prominent and should not be left unnoticed, even if both higher and lower values have been found in other studies (33, 36, 43). The first part of our research, which comprised about one-third of the inquiries, was conducted shortly after the second COVID-19 wave, a period marked by organizational turmoil and unstable work safety conditions. This timing may have influenced burnout outcomes.

The inverse association found between DP and O3I <4% partially favors our study hypothesis. Having in mind that DP scores range between 0 and 30, that DP levels are defined as high for a score of 10 or more points and that, in this study, the average DP score was 6.03, a variation of 11.132 points is clinically meaningful. This finding starts the discussion of O3I as a potential biological correlate of burnout, a research topic with a manifest paucity of evidence.

Two major questions arise from the outcomes of our study.

The first one is whether low levels of n3-FA are a risk factor to develop DP or a consequence of high DP individual levels – a question that cross-sectional designs cannot answer. Cohort studies, which are the most adequate to establish causality and evaluate the effect of multiple variables, would certainly shed more light on the roles biology and physiology play in burnout development. Associations between n3-FA and mental illness are generally portrayed in literature in a cause-effect direction, where membrane n3-FA induce changes in brain structure and function that may predispose or protect subjects from psychiatric diseases, rather than being damaged by the latest. Contrastingly, in burnout, physiological disfunction is usually seen as an aftermath of chronic workplace stress, reflected mainly in autonomous nervous system and hypothalamus-pituitary-adrenal axis derangements. This is also the assumption found in the extensive research that has looked into potential burnout biomarkers and the related “allostatic load” (61, 67, 69, 71–74). Similarly, studies who have analyzed structural and physiological changes in burned out individuals report reduced hippocampal volume as a response to chronic stress (102) and not as an individual characteristic predisposing to burnout. Reports on brain-derived neurotrophic factor inverse correlations with burnout were inconclusive in what concerns the cause-effect direction, as this protein is thought both to protect against stress-induced neuronal damage and to be lower in individuals exposed to prolonged stressful situations (26).

The second question is why this association is found specifically with DP and absent with EE and PA.

The three burnout dimensions are usually described as arising from a common work environmental dysfunction, usually correlated with each other (103) (which was also the case of our study), reflecting different perspectives of job overload consequences. Some authors have advocated that EE, DP and PA are conceptually different (104, 105) but these differences have been described mainly in a sociopsychological framework and not by virtue of different biophysiological pathways implicated in the genesis of each burnout dimension.

Our results raise the hypothesis that DP may have a stronger inner biophysiological foundation than EE or PA.

The term “depersonalization” is also used to describe a defensive coping strategy with which individuals limit their involvement and create distance with others (106). It is believed to result from pathological changes in the sensory system, body image and self-experience (107) and to be associated with several structural and functional brain alterations (108). DSM-5 considers the depersonalization disorder – also known as depersonalization-derealization disorder – an independent condition, describing it as a dissociative disorder characterized by persistent feelings of detachment and disconnection from reality. Some of the similarities that can be found between burnout DP and depersonalization disorder are that both involve feelings of detachment that can interfere with the sense of self and social functioning; both involve a sense of detachment or disconnection and can serve as coping mechanisms, albeit in different contexts; burnout DP helps individuals emotionally distance themselves from the stressors at work, while in depersonalization disorder, the detachment might serve as a defense mechanism against overwhelming emotions or trauma (124). In this sense it is arguable that DP may be “the most psychiatric” of the three burnout dimensions and the proximity to depersonalization-derealization disorder may be explained on the grounds of common or close biophysiological features. Further insights into the resemblances between depersonalization-derealization disorder and burnout-related depersonalization could potentially emerge from future functional imaging studies, especially considering that these techniques have already shown differences in brain region activation in studies of depersonalization-derealization disorder patients, such as decreased neural response in emotion-sensitive regions and increased response in areas involved in emotional regulation (109).

If our findings are replicated and supported by future studies, it is plausible that n-3 FA body levels may play a protective role against DP whereas EE and PA protection may depend almost exclusively on environmental factors, job characteristics and personality traits. Some authors have proposed DP as a prime component of burnout whose high levels result in EE and PA worsening (110), when the phenomenon is observed longitudinally. Under this point of view, protection against DP may have the potential to hamper the worsening of the other two dimensions and stickle overall burnout progression.

While this study does not specifically address interventions to prevent and mitigate burnout among healthcare workers, it is worth briefly highlighting some evidence-based strategies whose effects have been systematically evaluated. Individual-focused interventions include self-care workshops, stress management, communication skills training, yoga, massage, mindfulness, and meditation; structural or organizational interventions include workload/schedule rotation, stress management training, and Balint training (111). Psychotherapy and medication (antidepressants and anxiolytics) are commonly used in managing burnout by addressing symptom control. However, some authors view the use of antidepressants as an indication of severity and as a negative consequence of burnout (112, 113). Systematic reviews have suggested that combined interventions obtain greater improvements (114). Mindfulness based interventions (stress reduction programs, apps, meditation and training) either alone or combined with structural interventions have shown particularly promising in workers’ wellness improvement; as their effects tend to fade over time, mindfulness-based interventions need to be repeated for maintained efficacy in the long term (115). Future studies need not only to identify the best individual and organizational interventions capable of promoting wellness but also to evaluate its effect on patient care and on patient satisfaction (116). Our study has some limitations such as being single-centered, having a cross-sectional design and a convenience sampling method. Data on work environment characteristics, stress management behaviors, and workload intensity—factors known to influence burnout levels and potentially omega-3 levels—were not collected. The small number of subjects whose O3I was below 4% presents some hindrance to the depth and precision of our analysis. The latest could potentially be overcome in the future by tracking the evolution of subjects identified as at risk of burnout in larger samples.

If upcoming studies confirm O3I as a burnout biological correlate of clinical interest in practice, tests availability and access should be optimized as they are currently not a routinized analysis in laboratories.

## 5 Conclusions

In our study, an O3I below 4% was significantly associated with higher burnout DP levels. No associations were found between O3I and the other two burnout dimensions, EE and PA. These results contribute to expanding the knowledge of burnout’s biological correlates, an area previously identified as underdeveloped. Further observational and experimental evidence is needed to confirm or reject the association found between an O3I below 4% and higher DP and to clarify the reasons for differences among the three burnout dimensions. The inclusion of functional imaging studies in future research could potentially elucidate the different mechanisms involved in EE, DP, and PA. We suggest O3I should be included in prospective studies with larger samples that will investigate the evolution of several burnout biological correlates as the syndrome emerges and progresses in at-risk individuals, with a special focus on those with low O3I values.

## Data Availability

The raw data supporting the conclusions of this article will be made available by the authors, without undue reservation.
