# Supplementary material for: Association between omega-3 index and depersonalization among healthcare workers in a university hospital: a cross-sectional study
Source: Front Psychiatry. 2024 Nov 15;15:1425792. doi: 10.3389/fpsyt.2024.1425792 (PMC11604981; doi:10.3389/fpsyt.2024.1425792)
Supplement: Supplementary file 1 [file DataSheet1.docx]

**Analysis of the Linear Model’s Assumptions**

Dependent variable: Depersonalization

Independent variable: **O3I < 4%**, age, current depressive episode, DHA/EPA diet content and omega-3 supplementation


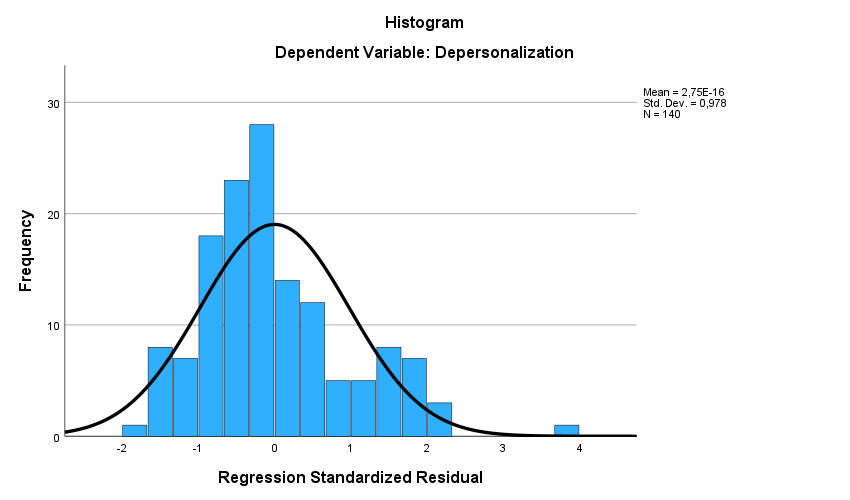


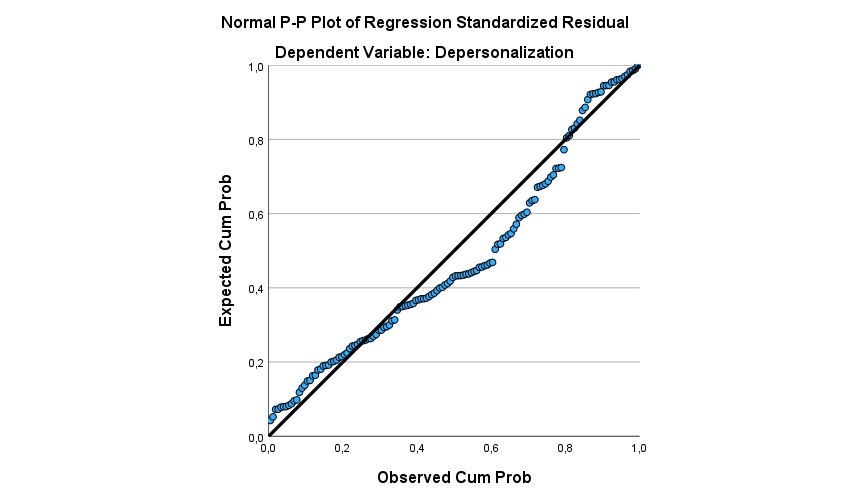


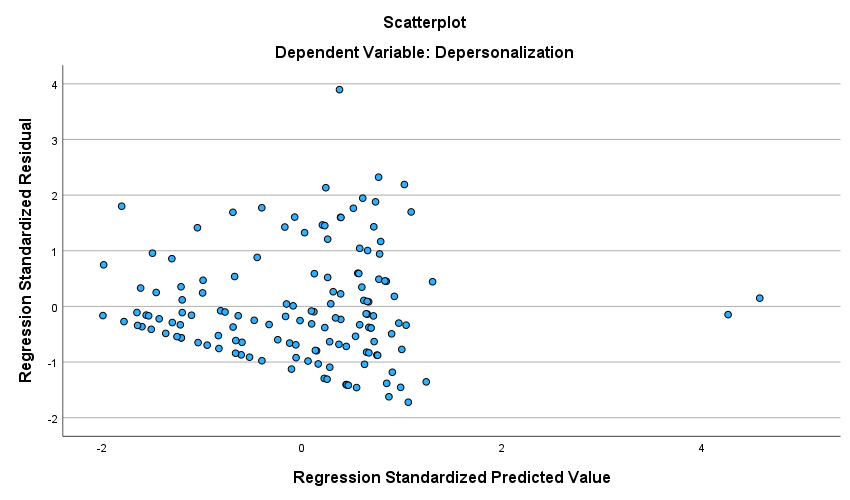


**Conclusions:**

- Normality is assumed based on residuals’ histogram and QQ-plot
- VIF is close to 1 for all the variables, except for DHA/EPA diet content (results were adjusted for these variables based on published evidence recommendations)
- While the graph of residual plots vs adjusted values’ pattern might suggest heteroscedasticity, the null correlation coefficient favours the model validity
