# Supplementary material for: Association between omega-3 index and depersonalization among healthcare workers in a university hospital: a cross-sectional study
Source: Front Psychiatry. 2024 Nov 15;15:1425792. doi: 10.3389/fpsyt.2024.1425792 (PMC11604981; doi:10.3389/fpsyt.2024.1425792)
Supplement: Supplementary file 2 [file Table1.docx]

Supplementary Material

# Supplementary Tables

| **Department of Origin, nº (%)** |  |
| --- | --- |
| Internal medicine | 34 (11.3%) |
| Emergency room | 79 (26.3%) |
| Surgery | 23 (7.7%) |
| Occupational Health | 20 (6.7%) |
| Pulmonology | 20 (6.7%) |
| Psychiatry | 13 (4.3%) |
| Anesthesiology | 8 (2.7%) |
| Cardiology | 8 (2.7%) |
| Intensive care | 6 (2.0%) |
| Neurology | 4 (1.3%) |
| Ear Nose and Throat | 4 (1.3%) |
| Gynecology | 3 (1.0%) |
| Other* | 78 (26.0%) |

**Supplementary Table 1.** Participants’ department of origin.

*Other departments: ambulatory consultation, cardiac/thoracic surgery, dermatology, endocrinology, gastroenterology, hematology, imagiology, immunohemotherapy, immunoallergology, laboratory medicine, nephrology, neurosurgery, oncology, ophthalmology, orthopaedics, pediatrics, pharmacology, plastic surgery, rheumatology, stomatology.

| **Doctors' Specialty, nº (%)** |  |
| --- | --- |
| Internal medicine | 14 (8.4%) |
| Work medicine | 14 (8.4%) |
| Surgery | 12 (7.2%) |
| Psychiatry | 12 (7.2%) |
| Pulmonology | 10 (6.0%) |
| Anesthesiology | 7 (4.2%) |
| Cardiology | 4 (2.4%) |
| Gynecology | 2 (1.2%) |
| Other* | 63 (37.7%) |
| No specialty | 29 (17.4%) |

**Supplementary Table 2.** Doctors’ specialties.

*Other specialties: cardiac/thoracic surgery, dermatology, endocrinology, gastroenterology, general practice/family medicine, hematology, imagiology, immunohemotherapy, immunoallergology, laboratory medicine, nephrology, neurosurgery, oncology, ophthalmology, orthopaedics, pediatrics, pharmacology, plastic surgery, rheumatology, stomatology.
